# Survival analysis of under-five mortality predictors: evidence from the 2011 and 2022/23 mozambique demographic and health surveys

**DOI:** 10.1186/s12889-026-27197-4

**Published:** 2026-04-25

**Authors:** Sancho Pedro Xavier, Audêncio Victor, Ana Raquel Ernesto Manuel Gotine, Manuel Mahoche, Patricia Hellen Rondó, Ageo Mário Cândido da Silva

**Affiliations:** 1 USP, School of Public Health, University of São Paulo (USP), Avenida Doutor Arnaldo, 715, São Paulo, 01246904 Brazil; 2 Institute of Collective Health, Federal University of Mato Grosso, Av. Fernando Correa da Costa, nº 2367 - Bairro Boa Esperança, Cuiabá, MT 78060-900 Brazil; 3 Faculty of Epidemiology and Population Health, London School of Hygiene & Tropical Medicine, Keppel Street, London, WC1E 7HT, London, UK

**Keywords:** Under-five mortality, Survival analysis, Risk factors, Mozambique

## Abstract

**Background:**

Under-five mortality (U5M) is a critical indicator for assessing the overall health status of a population. Mozambique faces challenges in this area, and understanding risk factors is essential for developing effective interventions. This study aimed to identify predictors of under-five mortality through survival analysis among children in Mozambique.

**Methods:**

This study used data from the 2011 (*n* = 7,647) and 2022/23 (*n* = 6,783) Mozambique Demographic and Health Surveys (MDHS), nationally representative surveys based on a multistage, stratified cluster sampling design. Under-five mortality was defined as the death of a live-born child before 60 months of age. Kaplan-Meier methods and Cox proportional hazards regression were used to estimate survival and identify predictors of mortality.

**Results:**

In 2011, 5.7% of children died before reaching age five and 4.7% within the first year of life. By 2022/23, these proportions had declined to 3.7% and 3.0%, respectively. A lower risk of mortality was observed among female children (2022/23: AHR = 0.56; 95% CI: 0.35-0.90), those living in households with five or more members (2011: AHR = 0.57; 95% CI: 0.43-0.76), and those in households with three or more children under five years of age (2011: AHR = 0.33; 95% CI: 0.18-0.61; 2022/23: AHR = 0.28; 95% CI: 0.13-0.61). In contrast, higher mortality risk was identified among children residing in urban areas (AHR = 1.42; 95% CI: 1.03-1.96), those reported to use bed nets for sleeping (2022/23: AHR = 2.39; 95% CI: 1.30-4.40), those with low birth weight (2011: AHR = 2.08; 95% CI: 1.30-3.33), and those delivered at home or outside public health facilities.

**Conclusion:**

These findings highlight the importance of strengthening maternal education and increasing investment in health systems to reduce under-five mortality in Mozambique. Expanding resources for maternal, neonatal, and child health services, particularly prenatal and obstetric care, is essential to further improve child survival outcomes.

**Supplementary Information:**

The online version contains supplementary material available at 10.1186/s12889-026-27197-4.

## Introduction

Under-five mortality (U5M) is a critical indicator of population health and a cornerstone of the third Sustainable Development Goal (SDG), which aims to reduce U5M to fewer than 25 per 1,000 live births by 2030 [1, 2]. Over the past three decades, there has been a significant improvement in infant survival worldwide [3, 4], providing better survival chances for millions of children compared to 1990 [2, 5]. Despite these advances, approximately 15,000 children die every day before reaching five years of age, including 2.5 million who die within their first month of life [6]. More than half of these deaths occur in Sub-Saharan Africa (SSA), with about 34% happening in the neonatal period [7]. However, it is estimated that up to 70% of these deaths could be prevented with effective prenatal care and safe childbirth [8, 9].

Globally, SSA is recognized as the region with the highest U5M rate [10, 11], with Mozambique being one of the countries with the highest rates worldwide [12]. Although U5M in Mozambique has drastically declined since 1990, with an estimated 79 deaths per 1,000 live births in 2015 [13], the country still faces significant challenges in this area [12]. The main causes of these deaths include complications related to pregnancy, pneumonia, diarrhea, neonatal sepsis, and malaria [14]. Moreover, factors such as maternal age, place of residence, education level, income index, sex of the child, birth order, birth weight, place of birth, attendance during childbirth, and the number of prenatal visits are also associated with the deaths of children under five years in these countries [15].

Worldwide, it is estimated that between 2016 and 2030, approximately 95 million children could die before the age of five if no further progress is made beyond the mortality levels observed in 2015. On the other hand, if countries sustain the pace of reduction in child mortality achieved between 2000 and 2015, the total number of deaths during this period could decrease to about 70 million [16]. To achieve the SDG targets of fewer than 25 deaths per 1,000 live births by 2030, and a total of 56 million deaths, most SSA countries need to significantly accelerate their progress [5].

A previous study in Mozambique examined factors associated with U5M using conventional regression approaches [17]. However, limited attention has been given to modeling the timing of death within the first five years of life while accounting for censoring, or to examining survival patterns across different Demographic and Health Surveys (DHS) rounds using a consistent analytical framework. Survival analysis provides a robust method for time-to-event data, allowing estimation of hazard over the risk period and improved understanding of survival patterns and associated risk factors. Therefore, this study aimed to identify predictors of under-five mortality in Mozambique using survival analysis applied to the 2011 and 2022/23 DHS datasets.

## Methods

### Study design and data collection

Secondary data analyses were conducted based on the Mozambique Demographic and Health Survey (MDHS) carried out from June to November 2011 and from July 2022 to March 2023. These two survey rounds were selected to examine temporal changes in under-five mortality and its predictors over an approximately ten-year period and to assess whether survival patterns and associated risk factors remained stable or changed over time. Both surveys are nationally representative and population-based, using standardized questionnaires and a multi-stage stratified cluster sampling design. The design included stratification by province and urban/rural residence, selection of Primary Sampling Units (PSUs) with equal probability, and selection of enumeration areas (EAs) proportional to size. Demographic and health data were collected from women aged 15 to 49 years and children under five years in the selected households. The dataset is available online through the link provided by the database: https://dhsprogram.com/data/dataset_admin/index.cfm.

### Population and sample size

The source population included all live births of children under five years old in the five years preceding the surveys conducted in the EAs. The children analyzed were born between 2006 and 2011 for the women interviewed in 2011, and between 2018 and 2023 for those interviewed in 2023. A total of 7,647 children in 2011 and 6,783 in 2022/23 were initially included in the analysis. The analysis was subsequently restricted to the most recent live birth among children under five years of age in the five years preceding each survey. The data were extracted from the birth record file (BR file) of the standard DHS dataset for Mozambique.

### Variables

The outcome variable considered was the time until death for children under five years old, expressed in months. The survival time of a child after 59 months was treated as censored (coded as 0), while children who died by 59 months were considered as events (coded as 1). Aligning with previous studies [1, 18–20], the independent variables analyzed included maternal obstetric and reproductive characteristics (mother’s age at first birth, current mother’s age, place of birth, type of birth, birth order, preterm birth, and number of children under five); socioeconomic and demographic factors (maternal education, mother’s marital status, type of residence, employment status, household size, household wealth, source of drinking water, type of cooking fuel, type of toilet, and mosquito nets in the home); maternal nutritional status (iron deficiency-anemia); and child characteristics (child’s sex), as outlined in Table 1. Household wealth was derived from the household wealth index. The original continuous wealth score was divided into tertiles based on the 33rd and 66th percentiles and recategorized into three groups (poor, middle, and rich). Source of drinking water, type of cooking fuel, and toilet facilities were recategorized into improved and unimproved categories [21, 22]. Variables including number of antenatal care visits, tetanus toxoid vaccination during pregnancy, birth attendant, and postnatal check within two days of delivery were excluded due to substantial missing data to ensure model stability.

**Table 1:** Analysis of factors associated with under-five mortality in mozambique, DHS 2011 and 2022/23

Predictors	Survival status at 59 months					
	2011			2022/23		
	Total n (%)	Dead n (%)	*p* – value *	Total n (%)	Dead n (%)	*p* – value *
Child's sex						
Female	2901 (48.5)	131 (4.4)	0.200	1624 (51.8)	50 (3.0)	0.100
Male	2996 (51.4)	157 (5.7)		1565 (48.2)	64 (5.0)	
Maternal age						
15-19	302 (13.9)	16 (5.2)	0.700	584 (17.6)	25 (5.0)	0.100
20-29	2926 (49.3)	143 (48.7)		1530 (49.4)	53 (3.7)	
30-39	2158 (30.0)	104 (36.8)		845 (25.7)	23 (2.7)	
40-49	511 (6.8)	25 (9.4)		230 (7.3)	13 (6.6)	
Marital status						
Married	4928 (84.9)	224 (4.7)	0.020	2543 (81.1)	96 (4.2)	0.200
Not Married	969 (15.1)	64 (7.0)		646 (18.9)	18 (2.6)	
Maternal education						
No education	2151 (37.1)	89 (39.4)	0.060	855 (29.8)	27 (3.8)	0.100
Primary	3062 (51.6)	169 (51.4)		1466 (47.2)	63 (4.5)	
Secondary or higher	684 (11.3)	30 (9.2)		868 (23.1)	24 (2.9)	
Household wealth						
Poor	1631 (34.9)	85 (4.8)	0.600	853 (33.3)	29 (3.7)	0.100
Middle	1931 (34.0)	90 (5.1)		1085 (33.3)	40 (4.7)	
Rich	2335 (31.2)	113 (5.3)		1251 (33.3)	45 (3.3)	
Type of residence						
Rural	3969 (72.2)	187 (7.6)	0.500	2142 (70.0)	85 (4.4)	0.070
Urban	1928 (27.8)	101 (7.0)		1047 (30.0)	29 (2.9)	
Currently employed						
No	3567 (59.0)	171 (5.0)	0.900	2127 (72.4)	80 (4.4)	0.300
Yes	2330 (41.0)	117 (5.1)		1062 (27.6)	34 (2.7)	
Source of water						
Improved	3305 (49.4)	170 (5.7)	0.400	2048 (58.2)	63 (3.3)	0.030
Not Improved	2592 (50.6)	118 (4.4)		1141 (41.8)	51 (4.9)	
Type of toilet						
Improved	1462 (21.2)	199 (6.1)	0.200	848 (26.9)	31 (4.3)	0.900
Not Improved	4435 (78.8)	612 (4.8)		2341 (73.1)	83 (3.8)	
Type of cooking fuel						
Improved	171 (1.9)	9 (5.7)	0.900	137 (3.5)	4 (3.1)	
Not Improved	5726 (98.1)	279 (5.0)		3052 (96.5)	110 (4.0)	0.600
Birth order						
1				779 (23.0)	35 (5.2)	
2-4	3680 (60.4)	174 (5.2)	0.400	1600 (51.0)	45 (3.0)	0.060
5 or more	2217 (39.6)	114 (4.9)		810 (26.0)	34 (4.6)	
Household size						
< 5	1585 (27.3)	130 (8.2)	< 0.001	906 (28.9)	53 (6.0)	< 0.001
5 or more	4312 (72.7)	158 (3.9)		2283 (71.1)	61 (3.1)	
Number of children under five						
< 3	4914 (84.4)	274 (5.7)	< 0.001	2708 (86.1)	105 (4.4)	0.060
3 or more	983 (15.6)	14 (1.4)		481 (13.9)	9 (1.0)	
Bednet for sleeping						
No	2140 (36)	112 (5.1)	0.500	1161 (39.5)	31 (2.3)	0.030
Yes	3757 (64)	176 (5.0)		2028 (60.5)	83 (5.0)	
Maternal age at first birth						
< 20	3931 (66.1)	205 (6.7)	0.080	2287 (71.3)	87 (4.1)	0.300
20 or more	1966 (33.9)	83 (7.8)		902 (28.7)	27 (3.5)	
Cesarean delivery						
No	5645 (96.2)	273 (5.0)	0.500	2281 (95.1)	81 (4.1)	0.800
Yes	252 (3.8)	15 (5.5)		157 (4.9)	5 (2.5)	
Maternal anemia						
No	2888 (47.0)	167 (4.5)	0.010	1727 (50.7)	51 (3.4)	0.030
Yes	3009 (53.0)	121 (5.6)		1462 (49.3)	63 (4.5)	
Place of delivery						
Home	2116 (41.9)	114 (5.4)	< 0.001	702 (26.7)	30 (5.2)	0.500
Others	121 (2.0)	13 (12.9)		791 (23.4)	29 (3.4)	
Private Sector	153 (2.6)	14 (9.9)		10 (0.2)	0 (0.00)	
Public Sector	3507 (53.5)	147 (4.2)		1686 (49.7)	55 (3.5)	
Birth weight						
Low weight	384 (6.2)	30 (7.1)	0.004	184 (6.7)	4 (2.0)	0.300
Normal	5513 (93.8)	258 (4.9)		2255 (93.3)	82 (4.2)	
Preterm Birth						
Not preterm	***	***	***	84 (2.2)	3 (3.4)	< 0.001
Moderate to late	***	***	***	3086 (97.3)	102 (3.7)	
Very preterm	***	***	***	19 (0.5)	9 (49.6)	

*Log-rank test *p*-value

***Variables were not collected during the study period

According to the WHO, a birth is considered preterm if it occurs before completing 37 weeks of gestation, and it is classified based on gestational age: extremely preterm (less than 28 weeks), very preterm (28 to less than 32 weeks), and moderate to late preterm (32 to less than 37 weeks) [23].

### Statistical analysis

Descriptive analyses were conducted for categorical variables, with absolute frequencies reported as observed counts and relative frequencies expressed as weighted percentages. The survival model was applied to analyze the time until the occurrence of an event, and a non-parametric model was used to handle censored data. Kaplan-Meier survival estimates and the Log-rank test were employed to investigate survival and mortality patterns in children under five years old [24]. The Kaplan-Meier estimates allowed us to graphically visualize the survival curves and estimate the median survival time [25, 26]. Additionally, the Tarone-Ware and Peto-Prentice tests were applied to assess differences between the survival curves [27]. Survival data are modeled in terms of two related functions: (i) the survival function $\:S\left(t\right)$ and (ii) the hazard function $\:h\left(t\right)$ [26]. The former represents the probability of children surviving beyond a time t from birth [28]. The latter indicates the probability of children under observation during time t experiencing the event, such as death. It represents the instantaneous rate of an event for an individual who has already survived up to time $\:t$ [26, 28].

The cumulative distribution function of $\:T$ is expressed as:

1 $$\:F\left(t\right)=P\left(T<t\right).$$

where 𝑡denotes the actual survival time of a child, $\:T$ indicates a random variable associated with survival time, and $\:F\left(t\right)$ is the probability density function for survival time. The distribution function 𝑇*T* and survival function $\:S\left(t\right)$ are expressed as:

2 $$\:F\left(t\right)=P\left(T>t\right)=\:\underset{0}{\overset{t}{\int\:}}f\left(u\right)du$$

3 $$\:S\left(t\right)=P\left(T\ge\:t\right)=1-F\left(t\right)$$

The hazard function is expressed as:

4 $$\:h\left(t\right)=\:\underset{\varDelta\:\to\:0}{\mathrm{lim}}\frac{P\:(t<T\:\le\:t+\:\varDelta\:t\backslash\:T>t}{\varDelta\:t}=\:\frac{f\left(t\right)}{S\left(t\right)}$$

where $\:\varDelta\:$ is the instantaneous change [18, 29].

The Cox hazard is modeled as follows:

5 $$\:h\left(t\right)=\:{h}_{0}\left(t\right)exp.\left({b}_{1}{X}_{1}+\:{b}_{2}{X}_{2}+\:{b}_{3}{X}_{3}+\cdot\:\cdot\:\cdot\:{b}_{k}{X}_{k}\right),$$

Where $\:{X}_{1}$ to $\:{X}_{k}$ are k independent variables and $\:{h}_{0}\left(t\right)$ is the baseline hazard at t, representing the risk for an individual with a value of 0 for all explanatory variables.

The hazard ratio (HR) is calculated by dividing both sides of Eq. (5) by $\:{h}_{0}\left(t\right)$ and taking the logarithm, as follows:

6 $$\:ln\:\frac{h\left(t\right)}{{h}_{0}\left(t\right)}=\left({b}_{1}{X}_{1}+\:{b}_{2}{X}_{2}+\:{b}_{3}{X}_{3}+\cdot\:\cdot\:\cdot\:{b}_{k}{X}_{k}\right),$$

Where $\:\frac{h\left(t\right)}{{h}_{0}\left(t\right)}$ is the hazard ratio. The coefficients $\:{b}_{1}$ to $\:{b}_{k}$ are estimated by Cox regression [30].

Both univariable and multivariable Cox proportional hazards regression models were used to examine the association between potential predictors and survival time through the hazard function. Variables showing statistical significance in the Log-rank, Tarone-Ware, and Peto-Prentice tests were included in the multivariable models. Hazard ratios (HRs) and corresponding 95% confidence intervals (CIs) were estimated. The proportional hazards assumption was assessed for each covariate and for the global model using Schoenfeld residuals [31], with results presented in the Supplementary Material (**S.** Figs. 2 and 3). In the final statistical model, maternal age was excluded due to collinearity. A two-sided p-value < 0.05 was considered statistically significant.

Survival probabilities and mortality incidence were estimated using the full eligible sample. Analyses of associations and Cox regression models were performed on the final analytic sample after excluding observations with missing predictor data. All analyses accounted for sampling weights, primary sampling units (clusters), and stratification variables using the survey package. Data analysis was performed using R software version 4.3.2 (https://www.r-project.org/) and RStudio version 2023.12.0 + 369 (https://posit.co/download/rstudio-desktop/).

## Results

### Sociodemographic characteristics of the study participants

When comparing the 2022/23 data with the 2011 results, similar trends as well as notable changes were observed (Table 1). The distribution of children under five by sex differed slightly between the two survey years, with females representing 51.8% in 2022/23 compared with 48.5% in 2011. There was a substantial increase in the proportion of mothers who began childbearing before age 20, rising from 66.1% in 2011 to 71.3% in 2022/23.

Regarding maternal education, although most mothers had primary education or no formal education in both years, there was a slight decrease in the proportion with primary education (from 51.6% to 47.2%) and in those with no formal education (from 37.1% to 29.8%). A modest improvement was observed in maternal anemia, which declined from 53.0% in 2011 to 49.3% in 2022/23. Changes were also evident in delivery practices. Although public health facilities remained the main place of delivery, their use declined from 53.5% to 49.7%, while home births decreased markedly from 41.9% to 29.7%.

The prevalence of low birth weight remained largely unchanged, at 6.2% in 2011 and 6.7% in 2022/23. Among preterm births, the vast majority were classified as moderate-to-late preterm (97.3%), whereas very preterm births remained rare (0.5%).

### Time to death in children under five by Kaplan-Meier estimates of the failure function

**Fig. 1:**
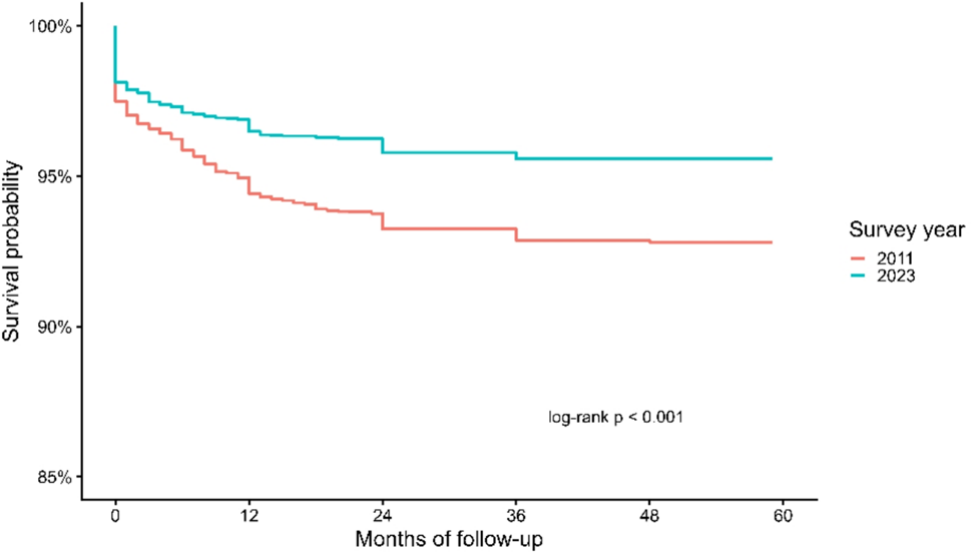
Kaplan–Meier estimates of the failure function for overall survival

A substantial difference in child survival between 2011 and 2022/23 was observed, and this difference was statistically significant (log-rank *p* < 0.001) (Fig. 1). In 2011, the unweighted probability of survival at 59 months was 92.8% (95% CI: 0.92-0.94), with a median time to death of 29.5 months (IQR: 14.75-4.25). The corresponding weighted estimate of survival at 59 months was 95.6%. The incidence of U5M was 5.6% (432/7,647). Of these deaths, 2.6% occurred within the first 30 days of life, 4.6% before one year, and 5.3% before 24 months. Weighted estimates were consistent, showing a U5M rate of 5.7% (SE 0.0032), with 5.4% (SE 0.0030) occurring before 24 months, 4.7% (SE 0.0030) before one year, and 2.5% (SE 0.0022) within the first 30 days of life (Supplementary Table 1 and Fig. 1).

In 2022/23, survival at 59 months improved to 95.6% (95% CI: 0.95-0.96) in the unweighted analysis, with no change in the median time to death. After applying sampling weights, the estimated survival probability remained similar. U5M declined to 3.6% (247/6,783), with a weighted estimate of 3.7% (SE 0.0029). Of these deaths, 1.9% (SE 0.0023) occurred within the first 30 days of life, 3.0% (SE 0.0027) before one year, and 3.5% (SE 0.0029) before 24 months (Supplementary Table 2 and Fig. 1).

**Table 2:** Crude and adjusted multivariable cox regression for child mortality in mozambique

	DHS 2011				DHS 2022/23			
Predictors	Crude model		Adjusted model		Crude model		Adjusted model	
	CHR (IC 95%)	*p* - value	AHR (IC 95%)	*p* - value	CHR (IC 95%)	*p* - value	AHR (IC 95%)	*p* - value
Child's sex								
Male	Ref		Ref		Ref		Ref	
Female	0.77(0.58-1.03)	0.079	0.79(0.57-1.05)	0.107	0.59(0.37-0.95)	0.028	0.56(0.35-0.90)	0.016
Marital status								
Married								
Not Married	1.43(1.02-2.00)	0.039	1.28(0.91-1.80)	0.162	0.58(0.32-1.07)	0.079	0.60(0.33-1.12)	0.108
Maternal education								
Primary	Ref		Ref		Ref		Ref	
Secondary or higher	0.91(0.57-1.44)	0.673	0.88(0.53-1.45)	0.617	0.63(0.36-1.10)	0.104	0.72(0.42-1.23)	0.231
No education	0.78(0.58-1.05)	0.105	0.81(0.60-1.09)	0.165	0.84(0.46-1.52)	0.567	0.88(0.45-1.74)	0.722
Household wealth								
Rich	Ref							
Middle	1.01(0.76-1.33)	0.961	0.84(0.63-1.12)	0.234	1.42(0.82-2.47)	0.211	**	**
Poor	1.13(0.85-1.50)	0.397	0.83(0.65-1.08)	0.164	1.15(0.65-2.01)	0.636	**	**
Type of residence								
Rural	Ref							
Urban	1.25(0.93-1.70)	0.145	1.42(1.03-1.96)	0.034	0.65(0.39-1.09)	0.101	0.64(0.38-1.10)	0.105
Birth order								
1			Ref		Ref		Ref	
2-4	Ref		**	**	0.57(0.32-1.03)	0.063	0.60(0.33-1.07)	0.084
5 or more	0.93(0.72-1.22)	0.618			0.89(0.49-1.62)	0.706	1.13(0.51-2.48)	0.765
Household size								
<5	Ref		Ref		Ref		Ref	
5 or more	0.48(0.36-0.62)	0.001	0.57(0.43-0.76)	< 0.001	0.52(0.32-0.85)	0.009	0.56(0.29-1.03)	0.063
Number of children under five								
< 3								
3 or more	0.27(0.15-0.48)	0.001	0.33(0.18-0.61)	< 0.001	0.24(0.11-0.50)	< 0.001	0.28(0.13-0.61)	0.001
Bednet for sleeping								
No	Ref							
Yes	1.02(0.76-1.36)	0.909	**	**	2.24(1.27-3.94)	0.005	2.39(1.30-4.40)	0.005
Maternal age at first birth								
>= 20	Ref		Ref		Ref			
< 20	1.18(0.88-1.59)	0.277	**	**	1.18(0.67-2.07)	0.563	**	**
Cesarean delivery								
No								
Yes	1.06(0.52-2.15)	0.869	**	**	**	**	**	**
Maternal anemia								
No	Ref		Ref		Ref		Ref	
Yes	1.29(0.98-1.71)	0.072	1.26(0.95-1.68)	0.107	1.34(0.83-2.17)	0.229	1.28(0.80-2.12)	0.293
Place of delivery								
Public sector	Ref		Ref					
Home	1.31(0.98-1.76)	0.063	1.69(1.20-2.38)	0.003	**	**	**	**
Private sector	2.43(1.21-4.86)	0.012	2.75(1.39-5.42)	0.004	**	**	**	**
Others	3.18(1.62-6.25)	0.001	3.53(1.77-7.05)	< 0.001	**	**	**	**
Birth weight								
Normal	Ref		Ref					
Low weight	1.50(0.97-2.33)	0.067	2.08(1.30-3.33)	0.002	**	**	**	**

***Variables were not collected during the study period

** Variables with a *p*-value > 0.05 were not included in the models

According to Table 1, in 2011, survival among females was 95.6% (*p* = 0.200). In 2022/23, female survival increased to 97.0% (*p* = 0.100). Survival varied by maternal education in both survey years. In 2011, children of mothers with no formal education had a survival rate of 90.6%, compared with 95.8% among those whose mothers had secondary or higher education (*p* = 0.060). By 2022/23, survival among children of mothers with no formal education increased to 96.2%, while it reached 97.1% among those whose mothers had secondary or higher education (*p* = 0.100).

Household size showed a consistent pattern, with lower survival in households with fewer than five members in both 2011 (91.8%) and 2022/23 (91.9%), compared with households with five or more members, where survival was 94.2% in 2011 and 94.0% in 2022/23 (*p* < 0.001).

Survival also varied by place of delivery. In 2011, survival was 95.8% among births in public health facilities and 94.6% among home deliveries. In 2022/23, survival was 96.5% for public-sector births and 94.8% for home deliveries. In 2011, children born with low birth weight had lower survival (92.9%) than those born with normal birth weight (95.1%) (*p* = 0.004). In 2022/23, survival among very preterm children was 50.4%, compared with 96.3% among those born moderate to late preterm (*p* < 0.001).

### Spatial distribution of survival in children under 59 months by province in Mozambique

**Fig. 2:**
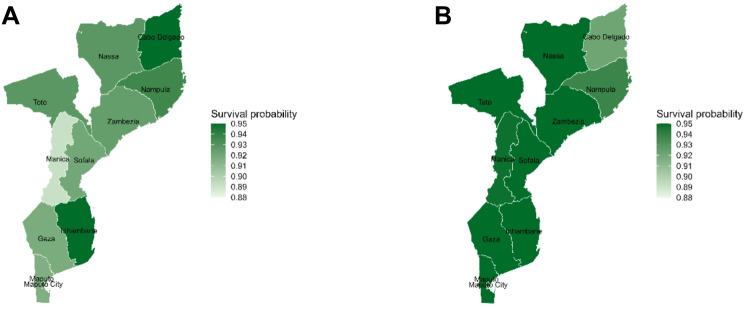
Spatial distribution of survival in children under 59 months. **A** represents survival distribution using data of 2011; **B** of 2022/23

According to Fig. 2, child survival in Mozambique showed substantial provincial variation between 2011 and 2022/23, with overall improvements across most provinces. Increases were observed in Gaza (from 91.8% to 95.2%), Maputo City (from 93.8% to 98.3%), Sofala (from 92.0% to 97.8%), and Tete (from 92.8% to 96.6%). Additional gains were seen in Manica (from 89.2% to 91.8%) and Zambezia (from 92.5% to 93.7%). In contrast, Niassa, Inhambane, and Cabo Delgado showed only minimal changes over the study period.

### Predictors of mortality in children under-five

Multivariable Cox regression identified several factors independently associated with U5M, as shown in Table 2. These included child sex, place of residence, household size, number of children under five in the household, bed net use, place of delivery, and birth weight.

In 2011, children residing in urban areas had a higher risk of U5M (AHR = 1.42; 95% CI: 1.03-0.96) compared with those living in rural areas. Living in households with five or more members was associated with a substantially lower risk (AHR = 0.57; 95% CI: 0.43-0.76), as was living in households with three or more children under five (AHR = 0.33; 95% CI: 0.18-0.61). Compared with public-sector deliveries, births at home, in private facilities, or in other locations were associated with a higher risk of U5M, with the greatest risk observed for deliveries in other locations (AHR = 1.69; 95% CI: 1.20-2.38; AHR = 2.75; 95% CI: 1.39-5.42; and AHR = 3.53; 95% CI: 1.77-7.05, respectively). Low birth weight was also associated with an increased risk of U5M (AHR = 2.08; 95% CI: 1.30-3.33).

In 2022/23, girls had a lower risk of U5M than boys (AHR = 0.56; 95% CI: 0.35-0.90). Children living in households with three or more children under five also had a lower risk (AHR = 0.28; 95% CI: 0.13-0.61). Bed net use was associated with a higher risk of U5M (AHR = 2.39; 95% CI: 1.30-4.40).

## Discussion

U5M remains a significant public health concern, contributing substantially to the deaths of children under the age of five and accounting for three-quarters of all mortality cases in SSA. Decreasing this rate is essential for attaining the United Nations SDGs [5]. This study, based on data from 2011 to 2022/23, observed significant differences in U5M rates, which decreased from 5.7% to 3.7% respectively. Various factors were identified as influencing U5M, including pregnancy-related complications, pneumonia, diarrhea, neonatal sepsis, and malaria [14]. Additionally, variables such as maternal age at first birth, place of residence, education level, wealth quintile, child’s sex, birth order, birth weight, place and mode of delivery, and number of prenatal visits were considered [15]. Predictors of U5M in this study included the child sex, place of residence, household size, number of children under five in the household, use of bed nets, place of delivery, and birth weight.

This study revealed that female children had a lower risk of mortality compared to male children. These findings are consistent with previous research conducted in Ethiopia [18, 20, 32, 33], Sierra Leone [34], Nigeria [19], and Tanzania [35]. This result is in line with scientific evidence indicating higher U5M rates in boys compared to female [36], potentially stemming from a complex interplay of external and biological factors. Boys are biologically more vulnerable and prone to diseases and premature death [37], with a heightened susceptibility to intrauterine growth restriction, preterm birth, and morbidities such as respiratory and gastrointestinal infections due to elevated testosterone levels that may suppress the immune system [38]. Additionally, the generally higher birth weight of boys compared to girls may contribute to complications during delivery and subsequent stages [10].

Place of residence was associated with the risk of U5M, with children of mothers living in urban areas presenting a higher risk of death. Although many studies report lower child mortality in urban setting [10, 39, 40], this advantage is not consistent across contexts and may change over time depending on epidemiological and socioeconomic conditions. In some periods, particularly in countries with a high HIV burden, children in large urban areas have experienced increased mortality [41]. This association may also reflect persistent urban inequalities, including overcrowding, barriers to quality healthcare, and greater exposure to air pollution. Air pollution is linked to prematurity, which in turn increases the risk of neonatal and under-five mortality [42, 43].

Our results showed that households with a greater number of members were associated with a lower risk of U5M, a finding consistent with a previously published study [44]. This association may be explained by the fact that larger households often include multiple caregivers, ensuring greater supervision of children and providing mutual support in situations of illness or economic hardship. Although some studies have reported an increased risk in larger households due to limited resources, in our study the social and caregiving advantages appear to have outweighed these potential disadvantages, resulting in a lower risk of U5M. Similarly, households with three or more children under five also showed a lower risk of U5M.

The use of bed nets for sleeping was unexpectedly associated with a higher risk of U5M. This finding contrasts with well-established evidence on the protective effect of bed nets against malaria [45], a leading cause of mortality in this age group [46, 47], and likely reflects contextual factors rather than a causal relationship. Reverse causality may play an important role, as households exposed to higher risks of illness or child mortality may be more likely to adopt preventive measures. In addition, previous research has shown that bed net use is not homogeneous within households and is less frequent in settings with multiple young children, which may result in misclassification when bed net use is assessed in aggregate. Ownership or self-reported use of bed nets may also serve as an indirect indicator of residence in malaria-endemic areas [48].

Low birth weight is a risk factor for U5M, supported by several previous studies [49–51]. This condition can be attributed to the immaturity of the baby’s organs, making them less capable of adapting to the external environment and independent life. Furthermore, most premature babies are more likely to contract sepsis, one of the leading causes of U5M [3]. Additionally, in this study, it was observed that children born very preterm had a significantly higher risk of death before reaching five years. This is because premature babies often face serious health problems at birth, which can threaten their survival [52].

The place of birth significantly influenced U5M, with children born at home, in the private sector, or other locations presenting a higher risk of mortality at five years compared to those born in public health institutions. Similar results were found in previous studies [1, 49, 51, 53–55], which highlighted the place of delivery as a significant predictor of child survival. However, some research found no association [33, 34, 56].

This relationship between place of birth and U5M can be explained by the crucial role of health facilities in promoting maternal and fetal health, which helps reduce delivery complications [53]. Another possible explanation is that giving birth in healthcare facilities can prevent pregnancy-related infant deaths. Moreover, babies born in these facilities receive the necessary care during delivery, as well as vaccination and guidance from healthcare professionals [51].

Limitations of this study include the use of secondary, cross-sectional DHS data, which restricts causal inference, and the reliance on self-reported information from mothers, which may be subject to recall bias. Although the 2011 and 2022/23 Mozambique DHS surveys employed standardized methodologies, minor differences in variable definitions, data collection procedures, and contextual factors, including changes in health policies, malaria control strategies, and socioeconomic conditions, may have influenced the observed trends.

Despite these limitations, this study addresses a major public health challenge in SSA and aligns with the SDGs. Our findings highlight key factors associated with under-five mortality, including child sex, household size, type of residence, number of children under five, place of delivery, birth weight, and malaria-related indicators. These results underscore the importance of improving maternal education and nutrition, strengthening malaria control efforts, and expanding access to quality maternal and child health services to enhance child survival and accelerate progress toward the SDGs.

## Conclusion

This study documents a substantial decline in child mortality in Mozambique, likely reflecting improvements in maternal education and access to skilled health care. Place of delivery and socioeconomic conditions emerged as central determinants of child survival, while the elevated risks associated with home births and low birth weight underscore persistent gaps in maternal and child health services. These findings highlight the need to strengthen antenatal, delivery, and postnatal care, with particular attention to reducing inequalities in access to obstetric and pediatric services. Sustained investment in these areas will be essential to consolidate gains and further advance child survival.

## Supplementary Information

Supplementary Material 1.

## Data Availability

In this study, datasets (MDHS 2011 e 2022/23, Mozambique) publicly available were analyzed. These data can be found at the following link: https://dhsprogram.com/data/dataset_admin/index.cfm upon registration and request.
